# Supplementary material for: Bacillus subtilis uses the SigM signaling pathway to prioritize the use of its lipid carrier for cell wall synthesis
Source: PLoS Biol. 2024 Apr 29;22(4):e3002589. doi: 10.1371/journal.pbio.3002589 (PMC11081497; doi:10.1371/journal.pbio.3002589)
Supplement: S1 Text — Fig A. Validation of the SigM-responsive reporter P(amj)-yfp. (A) Representative fluorescence images of the indicated B. subtilis strains harboring the σM-responsive reporter P(amj)-yfp after exposure to the indicated antibiotics for 30 min. Scale bar indicates 2 μm. (B) Quantification of images from the strains in (A). Bar represents median. (C) Quantification of fluorescence intensity from images of wild-type B. subtilis harboring the σM-responsive reporter P(amj)-yfp after exposure to the indicated antibiotics for 30 or 60 min as indicated. Bar represents median. The lack of full activation of σM by moenomycin is likely due to consumption of lipid II by the SEDS PG polymerases RodA and FtsW and generation of UndP. The data underlying B and C are provided in S1 Data. Fig B. GFP-SigM membrane localization depends on YhdLK and rapidly relocalizes to the nucleoid when UndP is sequestered. (A) Schematic of the reengineered SigM signaling system in strain BIR1100. A GFP-SigM fusion is expressed under the control of a xylose-regulated promoter. The SigM anti-sigma factors YhdL and YhdK are expressed under control of an IPTG-regulated promoter. The native sigM-yhdL-yhdK locus has been deleted (not shown). (B) Representative fluorescence images of the strain illustrated in (A). In the absence of inducers, there is faint GFP-SigM fluorescence. In the presence of 0.3 mM xylose, GFP-SigM localizes to the nucleoid. In the presence of both 0.3 mM xylose and 500 μM IPTG, GFP-SigM localizes to the membrane. (C) Representative time-lapse fluorescence images of the strain in (A) grown with 0.3 mM xylose and 500 μM IPTG before and after exposure to the indicated antibiotics. White carets highlight GFP-SigM localized to the nucleoid. Scale bars indicated 2 μm. Fig C. Antibiotics that block UndP recycling rapidly deplete the free carrier lipid pool. Representative images of wild-type cells treated with the indicated antibiotics for 2 min and then stained with fluorescently labeled MX [file pbio.3002589.s002.pdf]

Supplementary Information for:

***Bacillus subtilis* uses the SigM signaling pathway to prioritize the use of its lipid carrier for cell wall synthesis**

Ian J. Roney and David Z. Rudner

This PDF file includes:

Figures A to N
Tables A to C

Figure A
Roney and Rudner

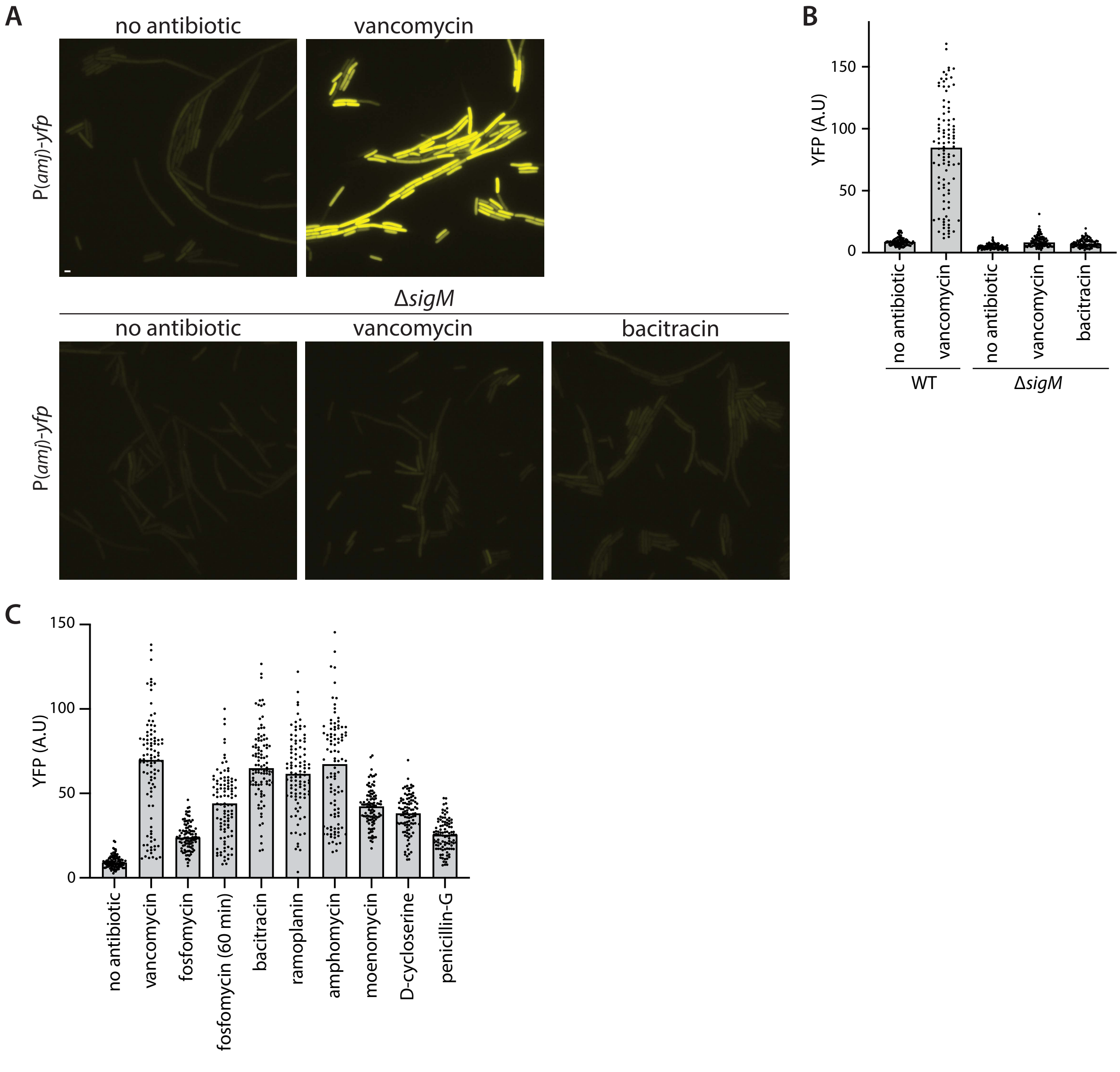

Figure A. Validation of the SigM-responsive reporter P(*amj*)-*yfp*. (A) Representative fluorescence images of the indicated *B. subtilis* strains harbouring the σ^M-responsive reporter P(*amj*)-*yfp* after exposure to the indicated antibiotics for 30 minutes. Scale bar indicates 2 µm. **(B)** Quantification of images from the strains in (A). Bar represents median. **(C)** Quantification of fluorescence intensity from images of wild-type *B. subtilis* harboring the σ^M-responsive reporter P(*amj*)-*yfp* after exposure to the indicated antibiotics for 30 or 60 min as indicated. Bar represents median. The lack of full activation of σ^M by moenomycin is likely due to consumption of lipid II by the SEDS PG polymerases RodA and FtsW and generation of UndP. The data underlying B, C are provided in S1 Data.

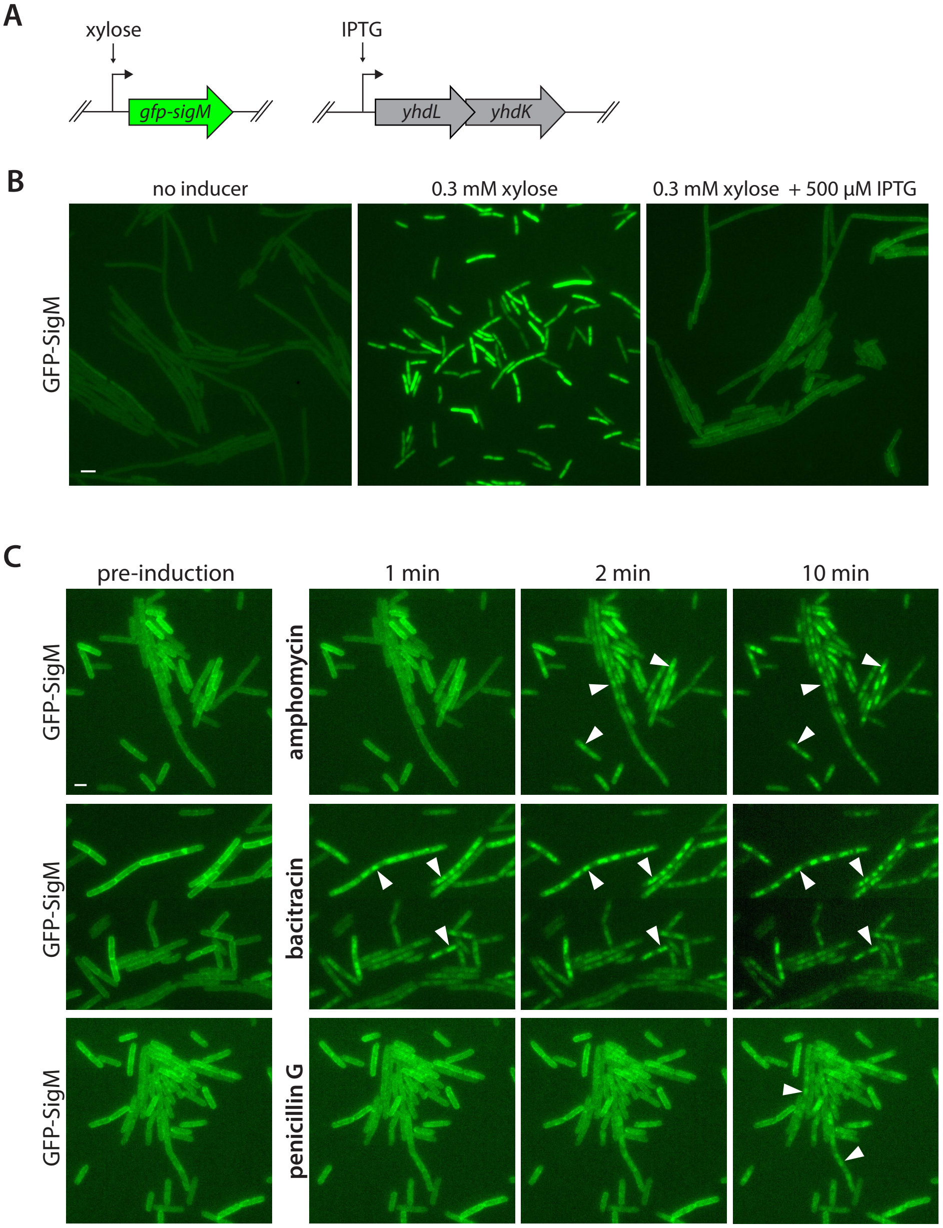

Figure B. GFP-SigM membrane localization depends on YhdLK and rapidly relocalizes to the nucleoid when UndP is sequestered. (A) Schematic of the re-engineered SigM signaling system in strain BIR1100. A GFP-SigM fusion is expressed under the control of a xylose-regulated promoter. The SigM anti-sigma factors YhdL and YhdK are expressed under control of an IPTG-regulated promoter. The native *sigM-yhdL-yhdK* locus has been deleted (not shown). **(B)** Representative fluorescence images of the strain illustrated in (A). In the absence of inducers, there is faint GFP-SigM fluorescence. In the presence of 0.3 mM xylose, GFP-SigM localizes to the nucleoid. In the presence of both 0.3 mM xylose and 500 µM IPTG, GFP-SigM localizes to the membrane. **(C)** Representative time-lapse fluorescence images of the strain in (A) grown with 0.3 mM xylose and 500 µM IPTG before and after exposure to the indicated antibiotics. White carets highlight GFP-SigM localized to the nucleoid. Scale bars indicated 2 µm.

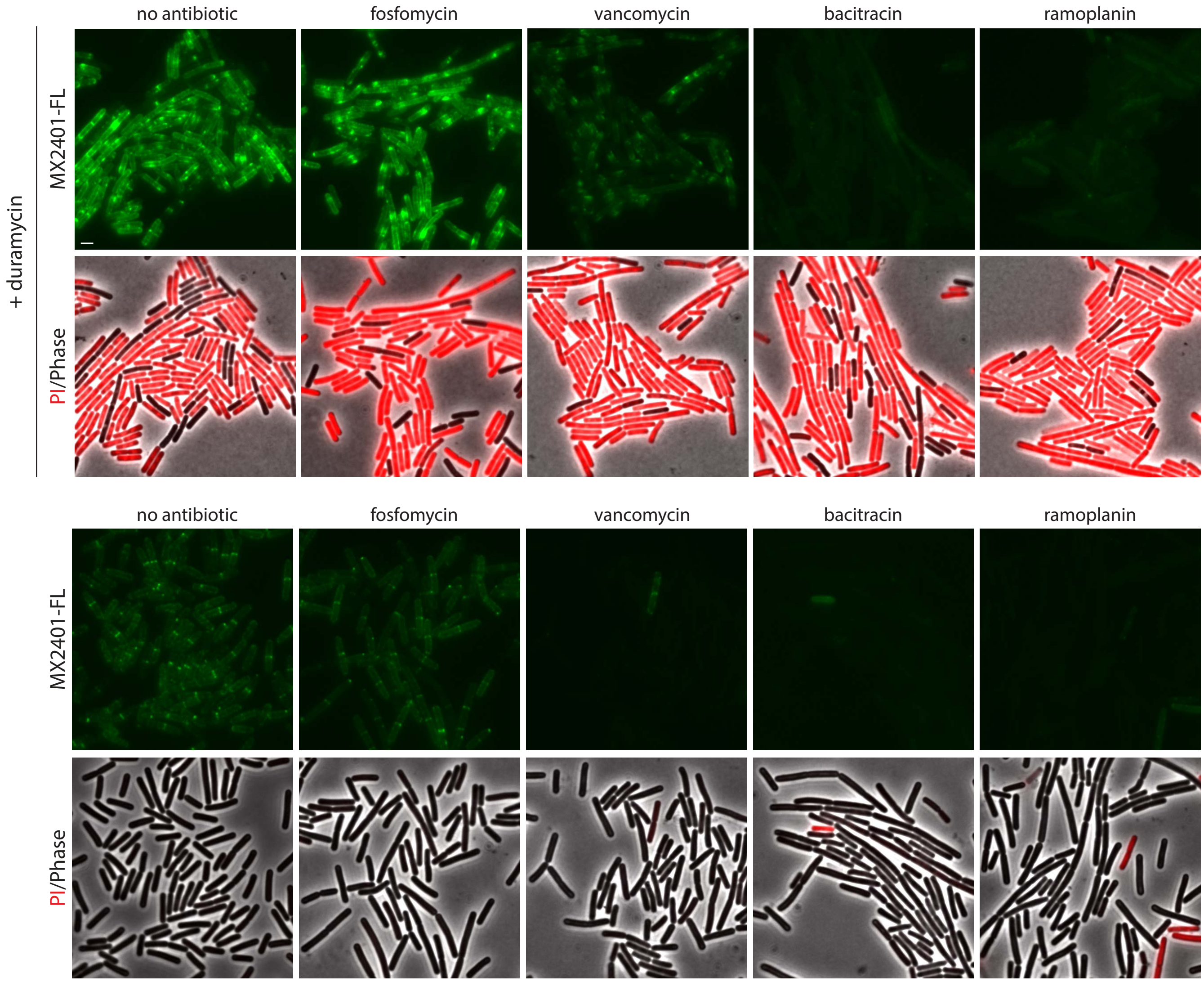

Figure C. Antibiotics that block UndP recycling rapidly deplete the free carrier lipid pool. Representative images of wild-type cells treated with the indicated antibiotics for 2 min and then stained with fluorescently labeled MX2401 (MX2401-FL) and propidium iodide (PI). Staining was performed in the presence and absence of duramycin. Duramycin generates pores in the membrane, allowing MX2401-FL to access inward-facing UndP in addition to outward-facing molecules. Phase-contrast and PI staining highlight cells with permeabilized membranes. Scale bar indicates 2 µm.

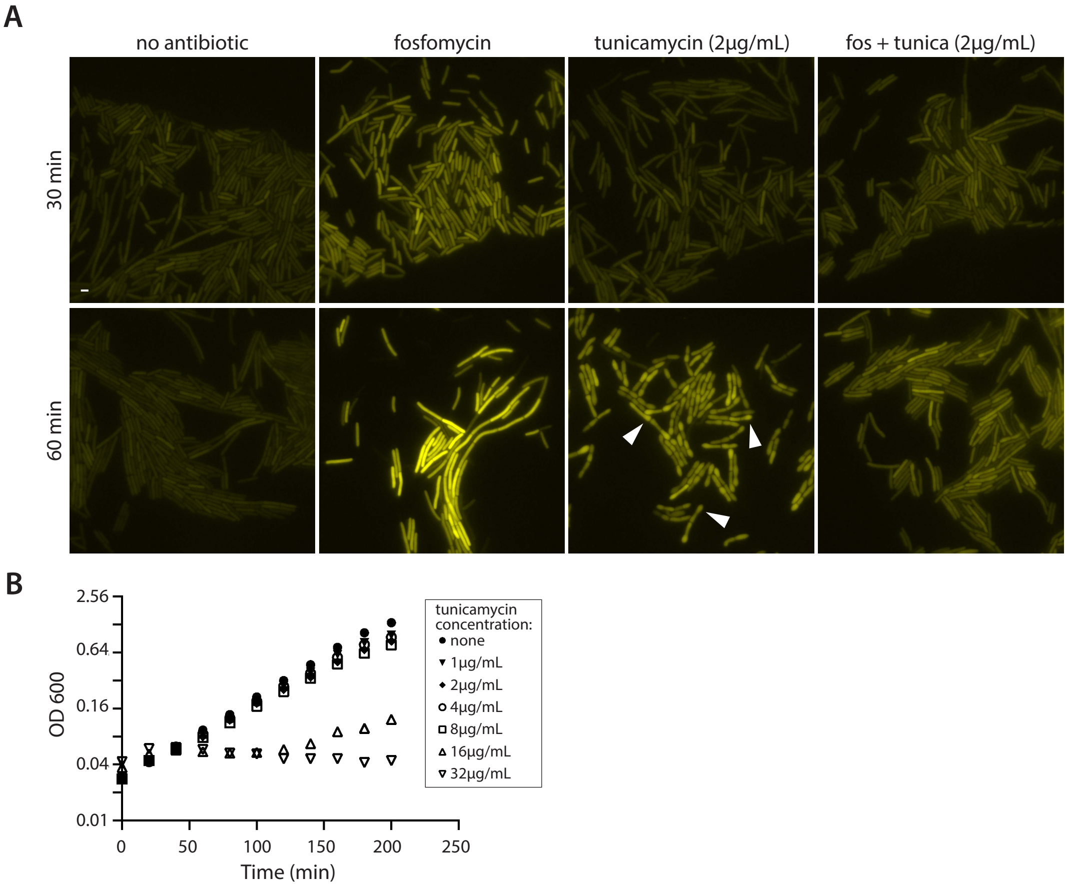

Figure D. Inhibition of wall teichoic acid synthesis suppresses fosfomycin-induced SigM activation. (A) Representative fluorescence images of wild-type *B. subtilis* cells harbouring a σ^M-responsive reporter (P(*amj*)-*yfp*) after exposure to the indicated antibiotics for 30 or 60 min. The tunicamycin concentration (2 µg/mL) used in this experiment inhibits TagO, the committing enzyme in wall teichoic acid synthesis, but not MraY, an essential enzyme involved in PG precursor synthesis. Carets highlight morphological defects associated with inhibition of wall teichoic acid synthesis. Scale bar indicates 2 µm. **(B)** Growth curves of wild-type *B. subtilis* cells in the presence of the indicated concentrations of tunicamycin. Tunicamycin has not impact on growth at 2 µg/mL. The data underlying B are provided in S1 Data.

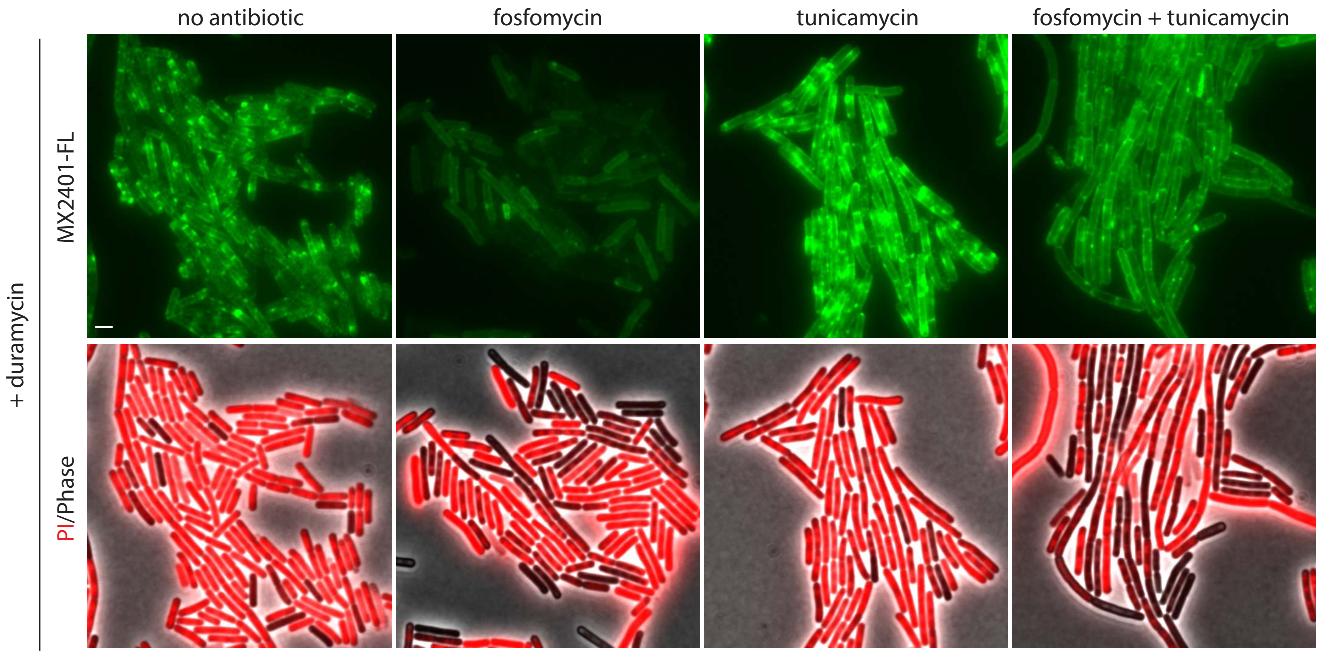

Figure E. Inhibition of wall teichoic acid synthesis suppresses the reduction in the free pool of UndP caused by fosfomycin. Representative fluorescence images of wild-type *B. subtilis* cells treated with the indicated antibiotics for 20 min and then stained with fluorescently labeled MX2401 (M2401-FL) and propidium iodide (PI). Staining was performed in the presence of duramycin to generate pores in the membrane allowing MX2401-FL to access both inward- and outward-facing UndP. Merged phase-contrast and PI images highlight cells whose membranes are permeable. The same MX2401-FL images are displayed in Figure 2C. Scale bar indicates 2 µm.

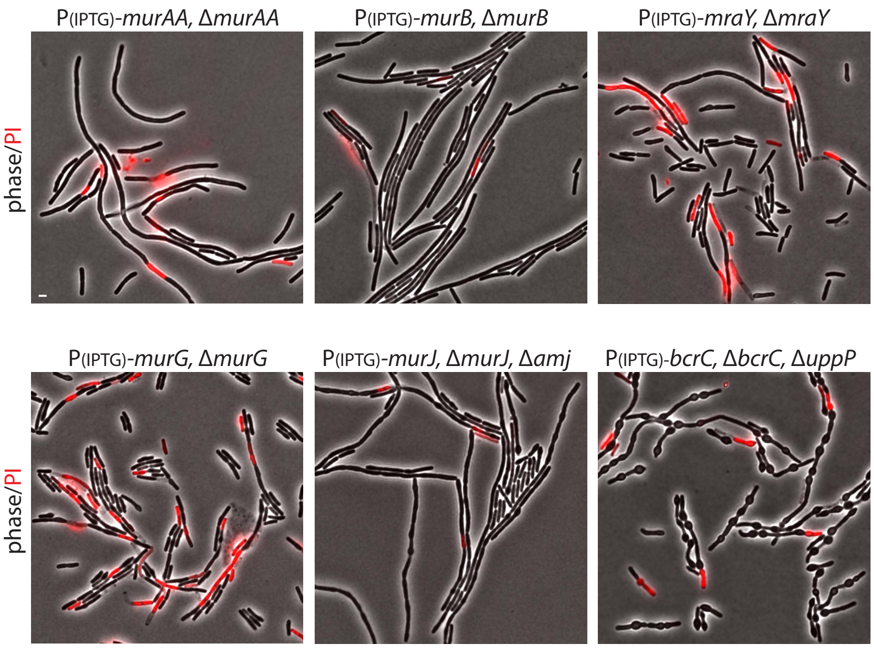

Figure F. Strains depleted of cell wall biogenesis factors have membrane integrity defects at the timepoint when SigM activity was analyzed. Representative phase-contrast images overlaid with fluorescence from propidium iodide labeling. Each strain was imaged at the same timepoint as indicated in Figure 3C. Scale bar indicates 1 μm.

Figure G
Roney & Rudner

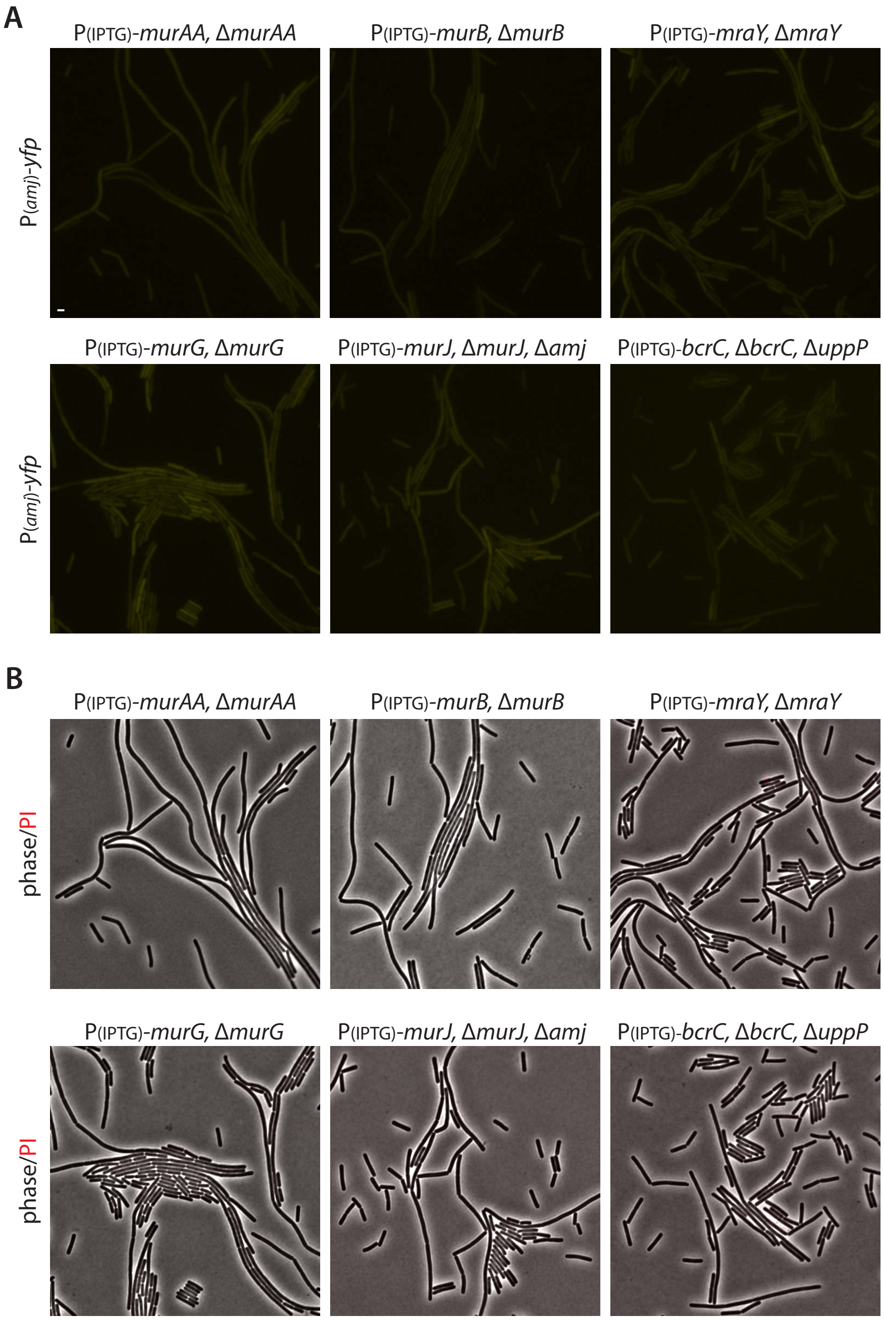

Figure G. Depletion strains have no morphological defects and do not activate SigM when grown under replete conditions. (A) Representative fluorescence images of the indicated *B. subtilis* strains harboring the σ^M-responsive reporter P(*amj*)-*yfp* grown in the presence of IPTG. Replete IPTG concentrations were 10 μM (*murAA*), 25 μM (*murB*), 25 μM (*mraY*), 12.5 μM (*murG*), 25 μM (*murJ*), 25 μM (*bcrC*). **(B)** Overlays of phase-contrast and propidium iodide staining of the same images in (A). Strains display no morphological defects, have intact membranes, and do not induce SigM. Scale bar indicated 1 μm.

Figure H
Roney & Rudner

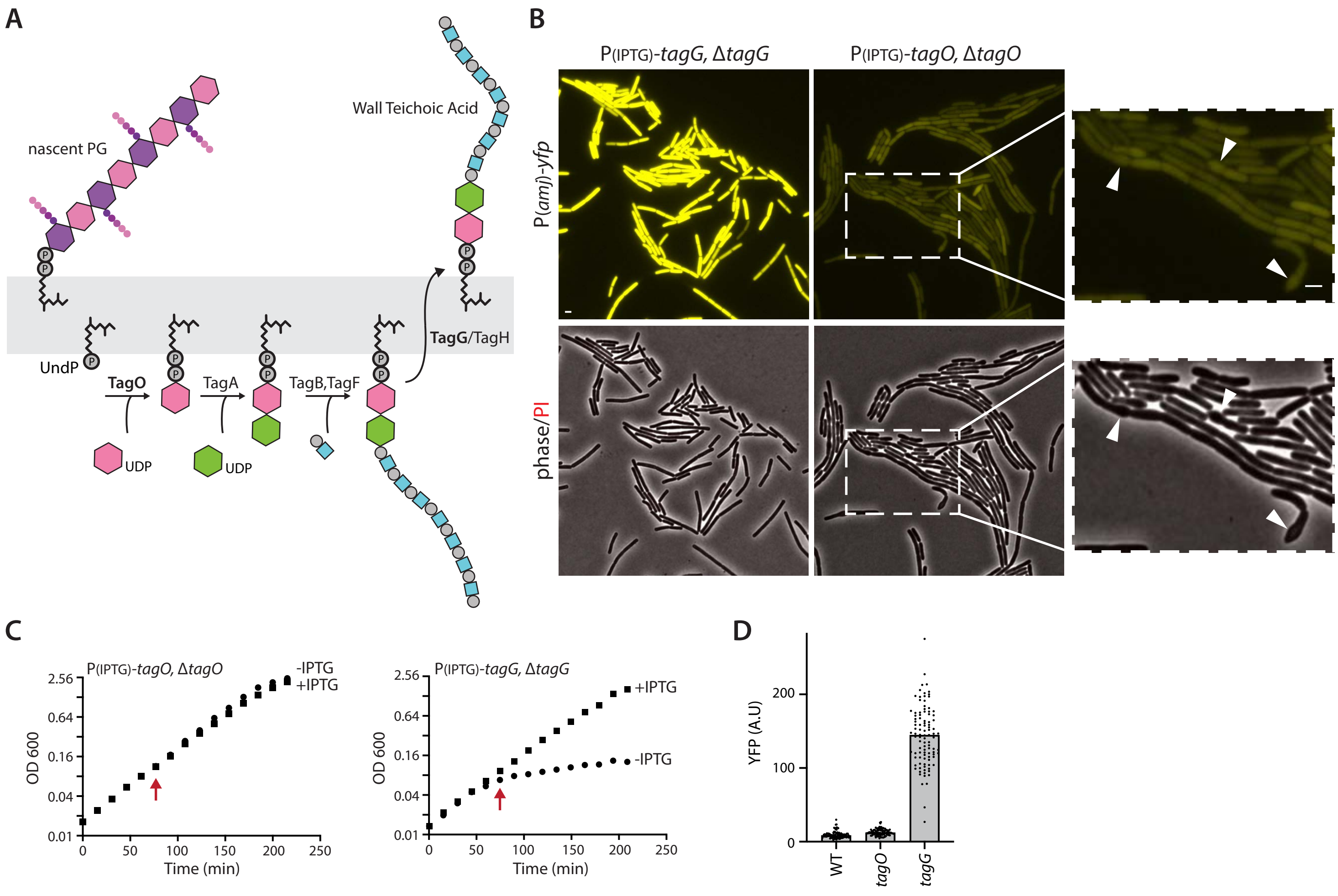

Figure H. Depletion of enzymes in the wall teichoic acid biosynthetic pathway that cause accumulation of UndP-linked intermediates activates SigM signaling. (A) Schematic of the wall teichoic acid biosynthetic pathway. Enzymes depleted are shown in bold. **(B)** Representative fluorescence and phase-contrast images of the indicated *B. subtilis* depletion strains harboring the σ^M-responsive reporter P(*amj*)-*yfp* after growth in the absence of IPTG. Inset highlights the morphological defects in the TagO depletion strain that do not cause SigM activation. Scale bars indicate 1 μm. **(C)** Growth curves of the indicated depletion strains grown in the presence (squares) or absence (circles) of IPTG. Red arrow indicates the timepoint at which samples were imaged. Permissive IPTG conditions were: *tagO* (12.5 μM), *tagG* (100 μM). **(D)** Quantification of YFP fluorescences from images as in (B). Bar represents median. The data underlying C, D are provided in S1 Data.

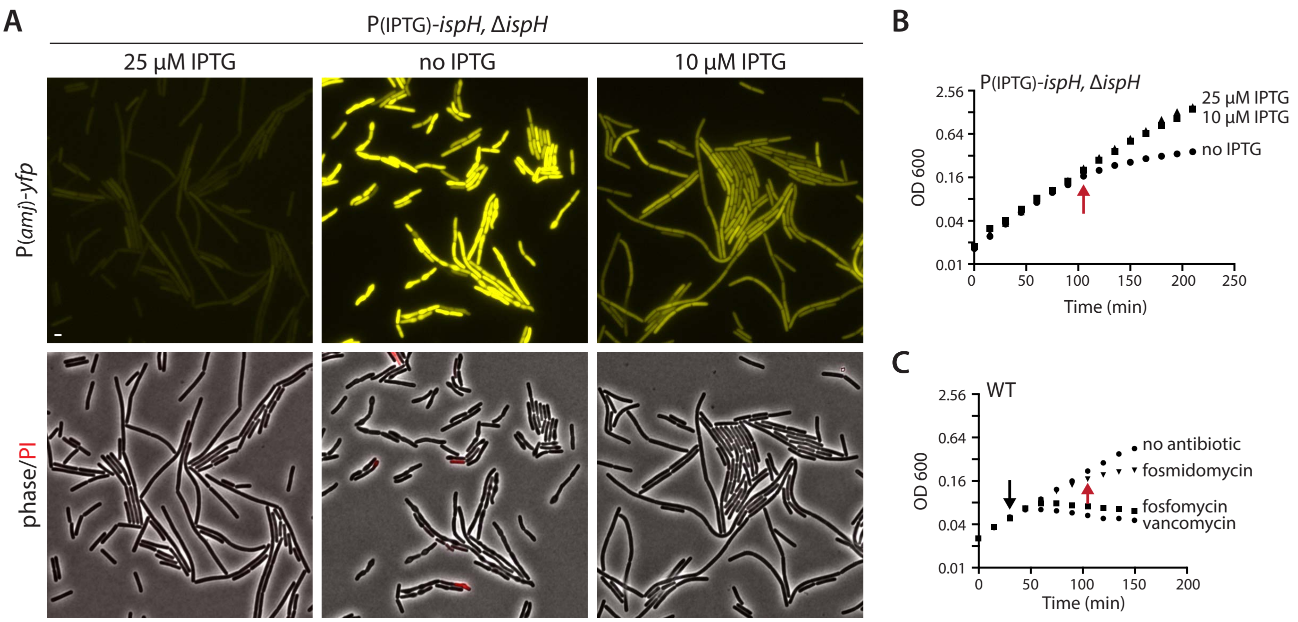

Figure I. Depletion of IspH activates SigM. (A) Representative fluorescence and phase-contrast images of the indicated *B. subtilis* IspH depletion strain harboring the σ^M-responsive reporter P(*amj*)-*yfp* grown in the presence or absence of IPTG. Even a partial depletion of IpsH (10 µM IPTG) causes SigM activation. **(B)** Growth curves of the depletion strain grown under replete (squares), partial depletion (triangles), or in the absence of IPTG (circles). Red arrow indicates the timepoint at which samples were imaged. **(C)** Growth curves of wild-type (WT) *B. subtilis* treated with fosmidomycin, vancomycin, or fosfomycin. Black arrow indicates the time when antibiotics were added. Red arrow indicates when sample was taken for imaging in Figure 4C. The data underlying B, C are provided in S1 Data.

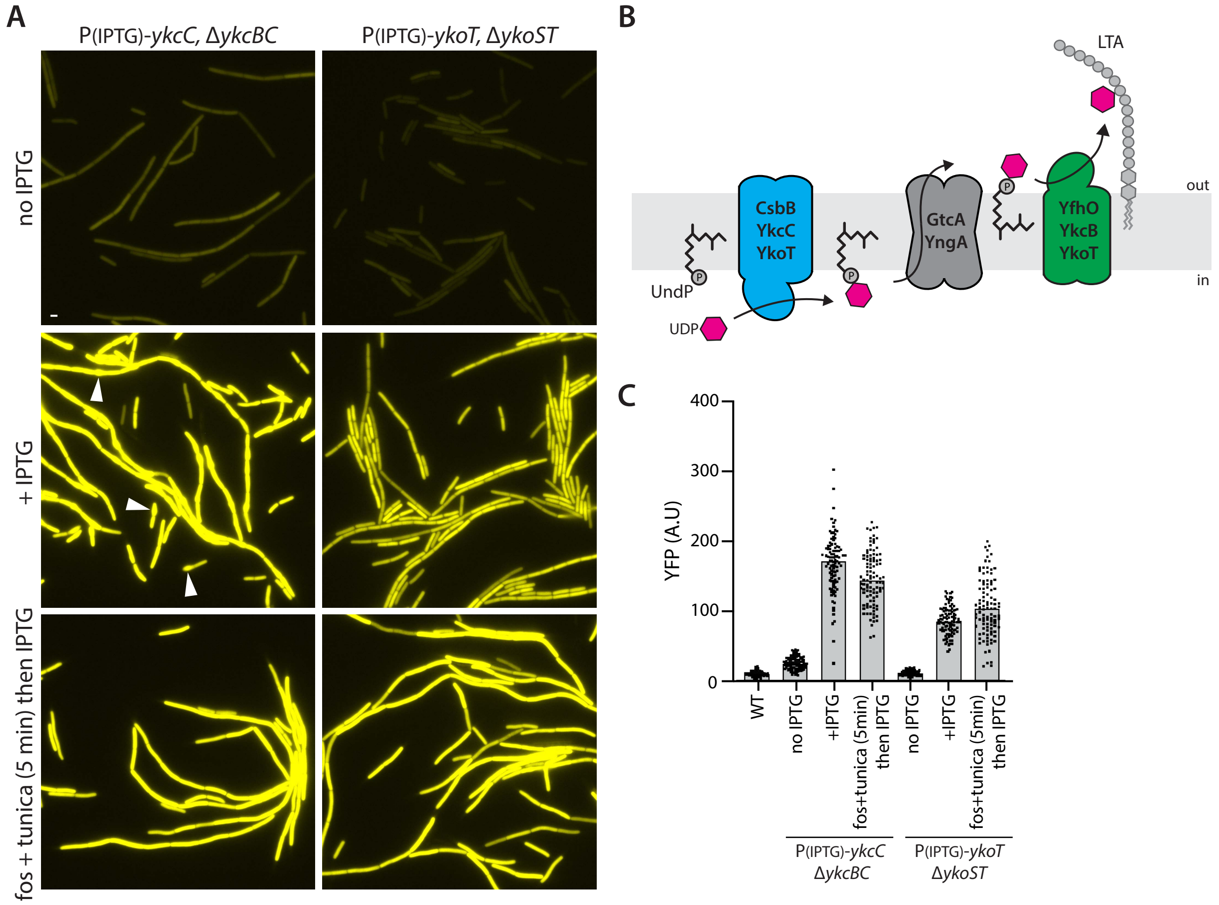

Figure J. Sequestering UndP-linked sugars in cell surface glycosylation pathways activates SigM. (A) Representative fluorescence images of the indicated *B. subtilis* strains harbouring the σ^M-responsive reporter P(*amj*)-*yfp*. Overexpression of YkcC or YkoT in the absence of YkcC or YkoT traps UndP-linked sugars and activates SigM. Carets highlight cells with morphological defects. Scale bar indicates 1 µm. **(B)** Schematic of cell surface glycosylation pathways. **(C)** Quantification of the YFP fluorescence in images similar to those in (A). Bar represents median. The partial suppression of SigM activation in cells pretreated with fosfomycin (fos) and tunicamycin (tunica) prior to IPTG addition is likely due to liberation of UndP from PG and WTA biogenesis pathways. The data underlying C are provided in S1 Data.

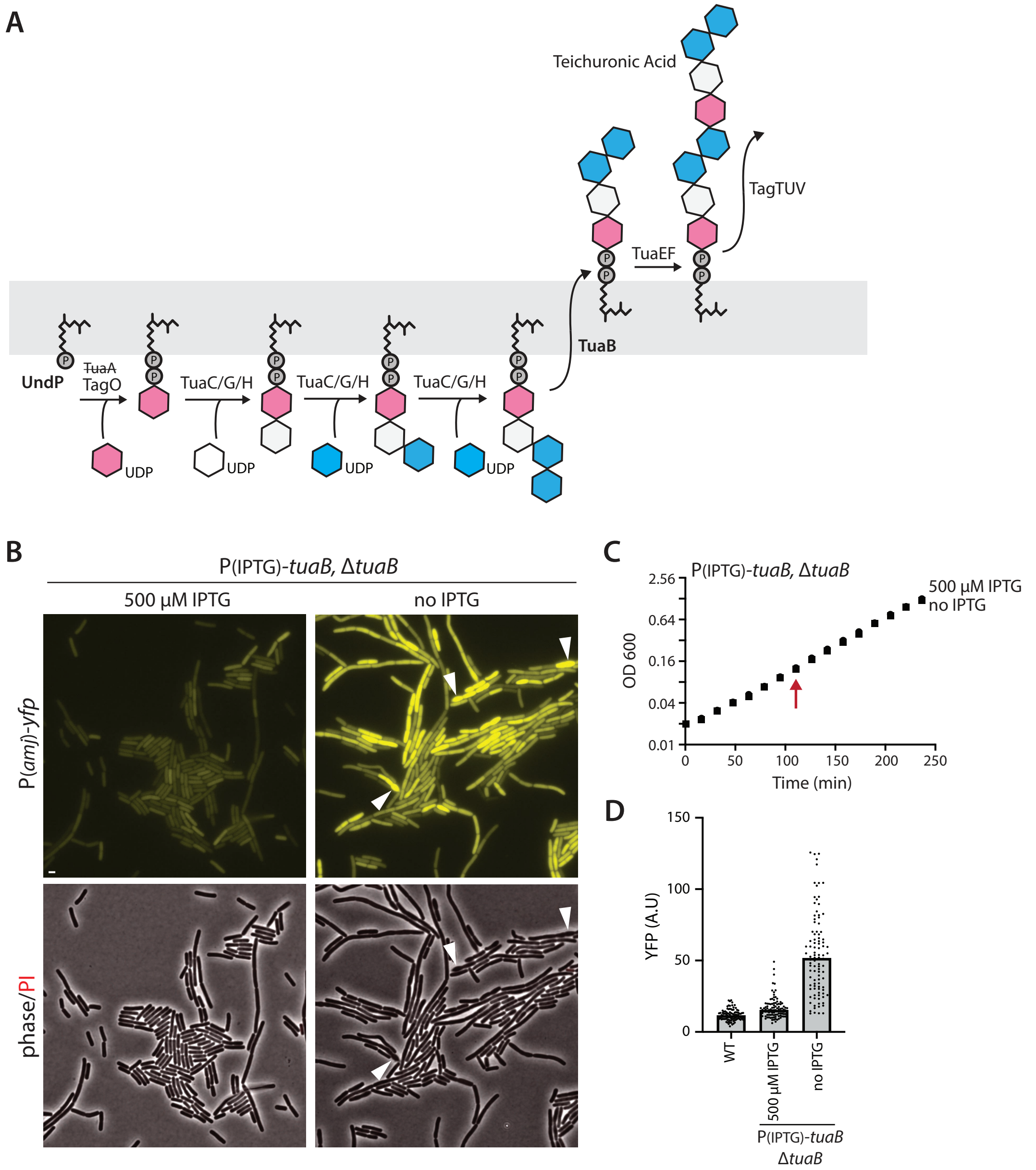

Figure K. Sequestering UndP in the teichuronic biosynthesis pathway activates SigM. (A) Schematic model of the teichuronic acid biosynthesis pathway. The *tuaA* gene in PY79 is a pseudogene and the committing step is thought to be catalyzed by TagO. The TuaB flippase is highlighted in bold. **(B)** Representative fluorescence and phase-contrast images of the indicated *B. subtilis* TuaB depletion strain harboring the σ^M-responsive reporter P(*amj*)-*yfp*. Carets highlight cells with morphological defects. Scale bar indicates 1 μm. **(C)** Growth curves of the TuaB depletion strain grown in the presence (squares) or absence (circles) of IPTG. Red arrow indicates the timepoint at which samples were imaged. **(D)** Quantification of YFP fluorescence from images in (B). Bar represents median. The data underlying C, D are provided in S1 Data.

Figure L
Roney & Rudner

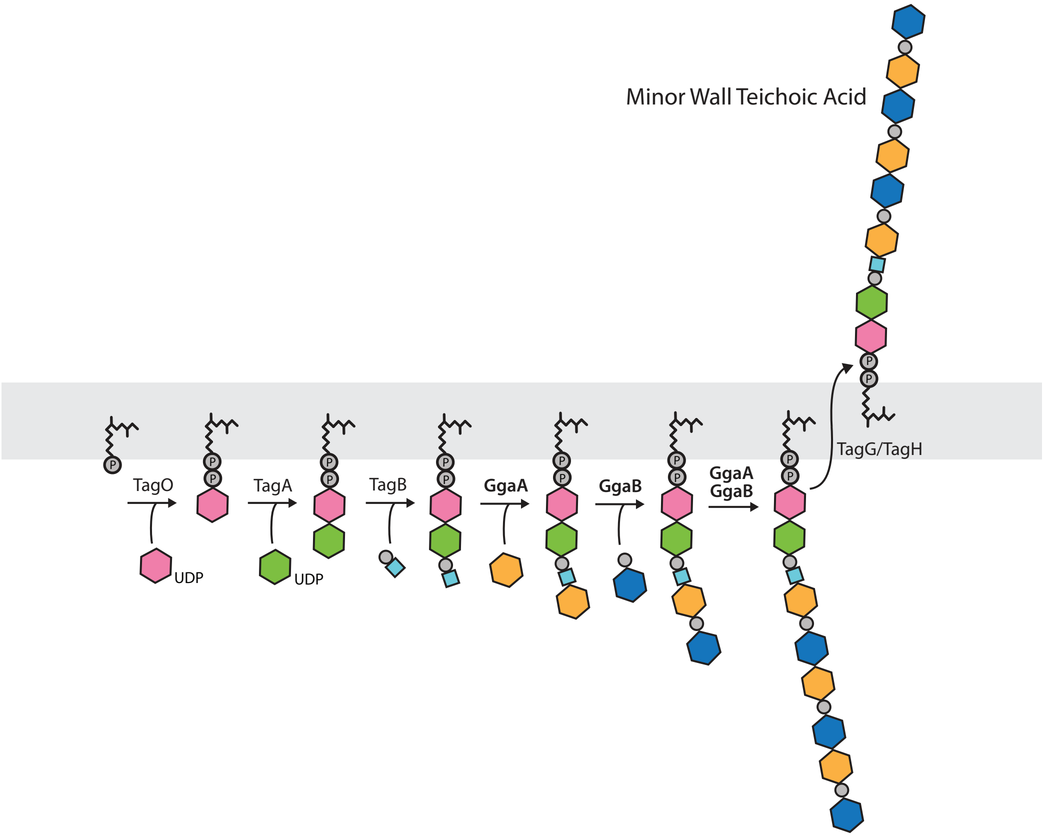

Figure L. Schematic of the minor teichoic acid biosynthesis pathway. Cells lacking GgaA exclusively make the major wall teichoic acid with polyglycerolphosphate. Cells lacking GgaB trap UndP in a minor teichoic acid precursor.

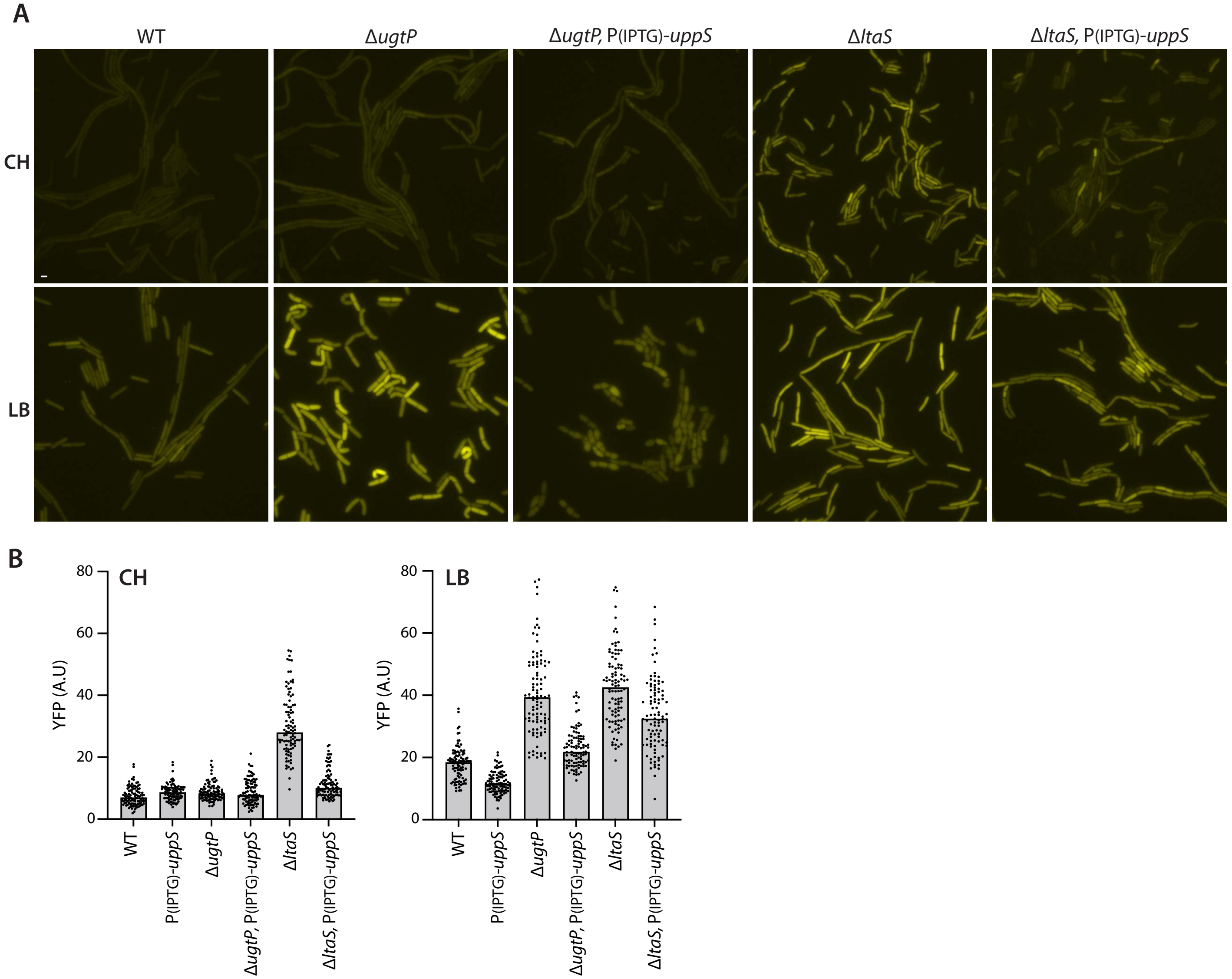

Figure M. Overexpression of UppS suppresses SigM activation caused by defects in the LTA synthesis pathway. (A) Representative fluorescence images of the indicated *B. subtilis* strains harboring the σ^M-responsive reporter P(*amj*)-*yfp*. Strains were grown in defined rich medium with casein hydrolysate (CH) or in Lysogeny Broth (LB). The strains with an IPTG-regulated allele of *uppS* were grown in the presence of 500 µM IPTG. Scale bar indicates 1 µm. **(B)** Quantification of the YFP fluorescence from images like those in (A). Bar represents median. Overexpression of UppS in a wild-type background reduced SigM activity when grown in LB, consistent with UndP directly modulating SigM activity. The data underlying B are provided in S1 Data.

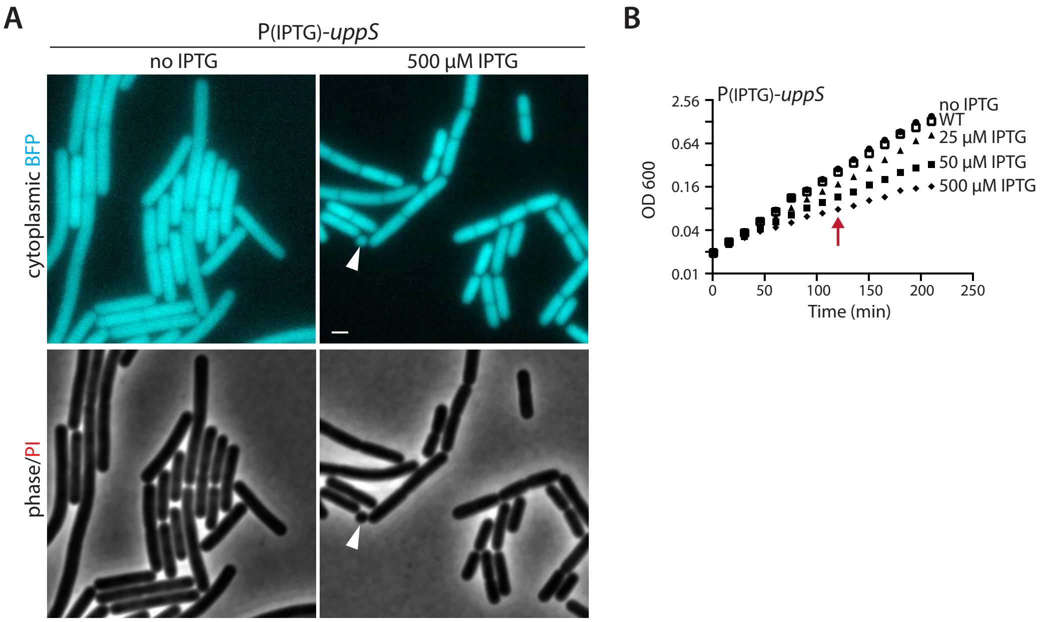

Figure N. Overexpression of UppS is toxic to *B. subtilis*. **(A)** Representative fluorescence and phase-contrast images of the indicated *B. subtilis* UppS overexpression strain harboring cytoplasmic BFP. Cells overexpressing UppS (500 µM IPTG) are shorter and occasionally form mini-cells (white caret). Scale bar indicates 1 µm. **(B)** Growth curves of UppS overexpression strain grown with different concentrations of IPTG. Red arrow indicates the timepoint at which cells were imaged in (A). The data underlying B are provided in S1 Data.

Table A. Strains used in this study

Strain	Background	Genotype	Source	Figures
BIR3	*B. subtilis PY79*	*wildtype*	(1)	1f, 2d, S1abc, S3, S5
BIR334	*B. subtilis PY79*	*sacA::Pveg-mTagBFP (phleo), amyE::Pamj-YFP (cat)*	This study	1bcd, 2a, 4cd, 5b, 7bc, S4ab, S9c, S10c, S11d, S13ab, S14ab
BIR592	*B. subtilis PY79*	*sacA::Pveg-mTagBFP (phleo), amyE::Pamj-YFP (cat), ycgO::Phyperspank-optRBS-uppS (spec)*	This study	S13ab, S14ab
BIR600	*B. subtilis PY79*	*sacA::Pveg-mTagBFP (phleo), amyE::Pamj-YFP (cat), ycgO::Phyperspank-optRBS-uppS (spec), ugtP::erm*	This study	S13ab
BIR603	*B. subtilis PY79*	*sacA::Pveg-mTagBFP (phleo), amyE::Pamj-YFP (cat), ycgO::Phyperspank-optRBS-uppS (spec), ltaS::erm*	This study	S13ab
BIR614	*B. subtilis PY79*	*sacA::Pveg-mTagBFP (phleo), amyE::Pamj-YFP (cat), ycgO::Pspank-tagG (spec), tagG::tet*	This study	S8bcd
BIR616	*B. subtilis PY79*	*sacA::Pveg-mTagBFP (phleo), amyE::Pamj-YFP (cat), ycgO::Pspank-murJ (spec), murJ::tet, amj::erm*	This study	3abc, S6, S7ab
BIR618	*B. subtilis PY79*	*sacA::Pveg-mTagBFP (phleo), amyE::Pamj-YFP (cat), ycgO::Pspank-optRBS-mraY (spec), mraY::Tet*	This study	3abc, S6, S7ab
BIR619	*B. subtilis PY79*	*sacA::Pveg-mTagBFP (phleo), amyE::Pamj-YFP (cat), ycgO::Pspank-murG (spec), murG::tet*	This study	3abc, S6, S7ab
BIR620	*B. subtilis PY79*	*sacA::Pveg-mTagBFP (phleo), amyE::Pamj-YFP (cat), ycgO::Pspank-optRBS-tagO (spec), tagO::Tet*	This study	S8bcd
BIR630	*B. subtilis PY79*	*sacA::Pveg-mTagBFP (phleo), amyE::Pamj-YFP (cat), ycgO::Pspank-murAA(spec), murAA:tet*	This study	3abc, S6, S7ab
BIR632	*B. subtilis PY79*	*sacA::Pveg-mTagBFP (phleo), amyE::Pamj-YFP (cat), ycgO::Pspank-murB(spec), murB::tet*	This study	3abc, S6, S7ab
BIR634	*B. subtilis PY79*	*sacA::Pveg-mTagBFP (phleo), amyE::Pamj-YFP (cat), ycgO::Pspank-uppS(spec), uppS::tet*	(1)	4bcd, 7b
BIR641	*B. subtilis PY79*	*sacA::Pveg-mTagBFP (phleo), amyE::Pamj-YFP (cat), ycgO::Pspank-bcrC(spec), bcrC::erm, uppP::tet*	This study	3abc,7b, S6, S7ab
BIR852	*B. subtilis PY79*	*sacA::Pveg-mTagBFP (phleo), amyE::Pamj-YFP (cat), ycgO::Pspank-ispH (spec), ispH::kan*	(1)	4d,7b, S9abc
BIR853	*B. subtilis PY79*	*sacA::Pveg-mTagBFP (phleo), amyE::Pamj-YFP (cat), ggaAB::tet, tuaABCDEFGH::kan*	This study	5b
BIR878	*B. subtilis PY79*	*sacA::Pveg-mTagBFP (phleo), amyE::Pamj-YFP (cat), ykcBC::tet, csbB-yfhO::erm, ykoST::kan*	This study	5b
BIR880	*B. subtilis PY79*	*ycgO::Pspank*-tuaB (erm), tuaB::tet, amyE::Pamj-YFP (cat), sacA::Pveg-mTagBFP (phleo)*	This study	7b, S11bcd

BIR893	*B. subtilis PY79*	*sacA::Pveg-mTagBFP (phleo), amyE::Pamj-YFP (cat), mlk::erm*	This study	7bc,S1ab
BIR894	*B. subtilis PY79*	*sacA::Pveg-mTagBFP (phleo), amyE::Pamj-YFP (cat), ycgO::Phyperspank-optRBS-ykcC (spec), ykcBC::tet*	This study	7c, S10ac
BIR895	*B. subtilis PY79*	*sacA::Pveg-mTagBFP (phleo), amyE::Pamj-YFP (cat), ycgO::Phyper-optRBS-ggaA(spec), ggaAB::tet*	This study	6ab
BIR901	*B. subtilis PY79*	*sacA::Pveg-mTagBFP (phleo), amyE::Pamj-YFP (cat), ycgO::Phyperspank-optRBS-csbB (spec), csbB-yfhO::erm*	This study	6ab ,7c
BIR902	*B. subtilis PY79*	*sacA::Pveg-mTagBFP (phleo), amyE::Pamj-YFP (cat), ycgO::Phyperspank-optRBS-ykoT(spec), ykoST::kan*	This study	7c, S10ac
BIR916	*B. subtilis PY79*	*sacA::Pveg-mTagBFP (phleo), amyE::Pamj-YFP (cat), ycgO::Phyperspank-optRBS-ykcC (spec), ykcBC::tet, mlk::erm*	This study	7c
BIR918	*B. subtilis PY79*	*sacA::Pveg-mTagBFP (phleo), amyE::Pamj-YFP (cat), ycgO::Phyperspank-optRBS-csbB (spec), csbB-yfhO::erm, mlk::kan*	This study	7c
BIR919	*B. subtilis PY79*	*sacA::Pveg-mTagBFP (phleo), amyE::Pamj-YFP (cat), ycgO::Phyperspank-optRBS-ykoT(spec), ykoST::kan, mlk::erm*	This study	7c
BIR944	*B. subtilis PY79*	*sacA::Pveg-mTagBFP (phleo), amyE::Pamj-YFP (cat), ugtP::erm*	This study	S13ab
BIR946	*B. subtilis PY79*	*sacA::Pveg-mTagBFP (phleo), amyE::Pamj-YFP (cat), ltaS::erm*	This study	S13ab
BIR1044	*B. subtilis PY79*	*sacA::Pveg-mTagBFP (phleo), amyE::Pamj-YFP (cat), ycgO::Pspank-uppS(spec), uppS::tet, mlk::erm*	This study	7b
BIR1097	*B. subtilis PY79*	*sacA::Pveg-mTagBFP (phleo), amyE::Pamj-YFP (cat), ycgO::Pspank-bcrC(spec), bcrC::erm, uppP::tet, mlk::kan*	This study	7b
BIR1098	*B. subtilis PY79*	*sacA::Pveg-mTagBFP (phleo), amyE::Pamj-YFP (cat), ycgO::Pspank-ispH (spec), ispH::kan, mlk::erm*	This study	7b
BIR1099	*B. subtilis PY79*	*ycgO::Pspank*-tuaB (erm), tuaB::tet, amyE::Pamj-YFP (cat), sacA::Pveg-mTagBFP (phleo), mlk::kan*	This study	7b
BIR1100	*B. subtilis PY79*	*ycgO::Pspank-LK (spec), mlk::erm, yvbJ::PxylA-sfGFP-sigM(kan)*	This study	1e, S2bc

References:

1. P. Youngman, J. B. Perkins, R. Losick, Construction of a cloning site near one end of Tn917 into which foreign DNA may be inserted without affecting transposition in Bacillus subtilis or expression of the transposon-borne erm gene. *Plasmid*. 12, 1–9 (1984).

2. I. J. Roney, D. Z. Rudner, Two broadly conserved families of polyprenyl-phosphate transporters. *Nature* 613, 729–734 (2023).

Table B. Plasmids used in this study

Plasmid	Description	Source
pGD24	ycgO::Phyperspank-uppS(spec) (amp)	This study
pIR086	ycgO::Pspank-yhdLK(spec) (amp)	This study
pIR175	ycgO::Phyperspank-csbB(spec) (amp)	This study
pIR176	ycgO::Pspank-mraY(spec) (amp)	This study
pIR177	ycgO::Pspank-tagO(spec) (amp)	This study
pIR190	ycgO::Pspank-tagG(spec) (amp)	This study
pIR192	ycgO::Pspank-murJ(spec) (amp)	This study
pIR193	ycgO::Pspank-murG(spec) (amp)	This study
pIR194	ycgO::Pspank-bcrC(spec) (amp)	This study
pIR209	ycgO::Pspank-murAA(spec) (amp)	This study
pIR210	ycgO::Pspank-murB(spec) (amp)	This study
pIR211	ycgO::Pspank-uppS(spec) (amp)	This study
pIR278	ycgO::Pspank-ispH (spec) (amp)	This study
pIR286	ycgO::Phyperspank-ykoT(spec) (amp)	This study
pIR287	ycgO::Phyperspank-ykcC(spec) (amp)	This study
pIR288	ycgO::Phyperspank-ggaA(spec) (amp)	This study
pIR344	yvbJ::PxylA-sfGFP-sigM(kan) (amp)	This study

Table C. Oligonucleotides used in this study

Name	Sequence
oGD87	CCGAATTAGCTTGCATGCgaGCTAGCctaAATTCCGCCAAACCTCC
oGD88	GTGAGCGGATAACAATTAAGCTTacaTAAGGAGGaactactatgCTCAACATACTCAAAAATTGG
oIR078	GTTGACCAGTGCTCCCTGAGCCGAGCTTTAATTTTTTCTG
oIR079	CCAAAATCTTTCTCGTCTGG
oIR153	ATTGTGAGCGGATAACAATTAAGCTacataaggaggaactactATGATG
oIR159	GCatCTGCAGttACTAGTtaCCCGGTTAAAAGCCAAACTGTAAATTCG
oIR334	ACAATTAAGCTTacataaggaggaactactATGCTTGACGAACGCATGATTC
oIR335	GCAGttACTAGTTTAATTCCTTTTCACCAGCCG
oIR336	ACAATTAAGCTTacataaggaggaactactATGCTTGAGCAAGTCATTCTG
oIR337	GCAGttACTAGTTTATAACCACACCTCGATGTAAATTC
oIR338	ACAATTAAGCTTacataaggaggaactactATGAAGCAAGGATTAATCTCG
oIR339	GCAGttACTAGTTCAGTGCATTTTTGTCAGACG
oIR340	TGATTGCTCAACGGATGGGACC
oIR341	CTGAGCGAGGGAGCAGAAGGAAAGAAGTCTCCTTTGGAATTAAATG
oIR342	GTTGACCAGTGCTCCCTGAGAAACTCCGGCTTATGTGCCG
oIR343	CTATCTACTCTTTATGATACCAAAATACG
oIR344	GAAGTTCCGAATGTTGTAGGG
oIR345	CTGAGCGAGGGAGCAGAAGGTGTCCTTTTCTCCTCCTGTTTC
oIR346	GTTGACCAGTGCTCCCTGAGGTGGAAAATGATCAATTTTTACAG
oIR347	CCGATCAGCCCGAGAAAGAC
oIR372	TTGTGAGCGGATAACAATTAAGCTTcaattaagtctaaggaagataaaaaatg
oIR373	GCgaGCTAGCatCTGCAGttACTAGTttaaagaaagtcaacaaacttgtctc
oIR374	GATAACAATTAAGCTTgtctactaaatataaaaatgtaaaaggtg
oIR375	TGCAGttACTAGTttagaaattttgatcggttggtttttc
oIR384	ctcaattgattcagaacacc
oIR385	CGGTACTGAGCGAGGGAGCAGAAtttttatcttccttagacttaattgtttttg
oIR386	CGGTAGTTGACCAGTGCTCCCTGtacgtaaggagattttacgatg
oIR387	gatgagccgtttgcgtttctg
oIR388	cgttaccgaactgtcttcg
oIR389	CGGTACTGAGCGAGGGAGCAGAAttctttttcccccagtctaag
oIR390	CGGTAGTTGACCAGTGCTCCCTGtagaaaagcaaatggaatgcgg
oIR391	ccggtatgtatgacgcatcttc
oIR419	gcataggaagtagagattttgg
oIR420	CGGTACTGAGCGAGGGAGCAGAAgattttgattctccccattagac
oIR421	CGGTAGTTGACCAGTGCTCCCTGgtcgaaaaataaggctcttttcc
oIR422	ggcagcagcatcagtttaatag
oIR423	cggttttgtagaagtccgtcc
oIR424	GAACGGTAGTTGACCAGTGCTCCCTGggtattcccctccgcgtccctaaattc
oIR425	CGGTACTGAGCGAGGGAGCAGAAacttacttgaaaatcagtatgtcac
oIR426	caaaagaagatttcgcctcgttc
oIR427	cagagtttatgaagcttgcg
oIR428	CGGTAGTTGACCAGTGCTCCCTGcgtaaacctccgcattcc
oIR429	CGGTACTGAGCGAGGGAGCAGAAttcaagttctgactgaagctgttc
oIR430	ggagttgagttcatttatcagc
oIR435	GGAATTGTGAGCGGATAACAATTAAGCTTgaatttagggacgcggagg
oIR436	CATGCgaGCTAGCatCTGCAGttACTAGTttatgcatttaagtcagaaacgac
oIR437	TAACAATTAAGCTTgcaaatggaatgcggaggtttac
oIR438	CTGCAGttACTAGTtcagcgatttccgccgatg

oIR439	TTGTGAGCGGATAACAATTAAGCTTaaccttttgggtgacggag
oIR440	CATGCgaGCTAGCatCTGCAGttACTAGTctaaattccgccaaacctccg
oIR657	ACTAGTAGAGCCGCTgccggtgctAC
oIR697	gaatgAAGCTTgaGCTAGCatCTCGAGAacataaggaggaactactATGAAGGGAGAAGAGTTGTTTACGGGTG
oIR710	GATTTTCGTTTATATCATATCAACCC
oIR711	CGGTACTGAGCGAGGGAGCAGAAGTTTGTACCTCTTTATTTAGAAGTTAAAGG
oIR716	CGGTAGTTGACCAGTGCTCCCTGGGTTGGTTTGTTTTATATTGACACTTC
oIR717	GAATAGTTTAACCATAAATTTTTTCGATC
oIR747	CACTATAAATTTCGTGAAATTCCAC
oIR748	CGGTACTGAGCGAGGGAGCAGAAGTAAATTCACCTCAATGTAATCAAC
oIR749	CGGTAGTTGACCAGTGCTCCCTGTGGCGTACTTGAGAGCATACG
oIR750	CATGAAGGAGCTAGAGTTCGAAGAC
oIR761	CGTACACTTCTTCAAGGTACGTATAAAGC
oIR762	CGGTACTGAGCGAGGGAGCAGAATTATTTTTCACTCCTTTTTGTCTAACTTTGAAATAG
oIR763	CGGTAGTTGACCAGTGCTCCCTGCCTCAAACCCCCTGTCCGTAATG
oIR764	GATGAAAACAGAACGAAAGGTAATGAG
oIR765	GCCACGGAGGACAATTTTTCTAACC
oIR766	CGGTACTGAGCGAGGGAGCAGAATGCTTACACATCCATTTGTTATTCTG
oIR767	CGGTAGTTGACCAGTGCTCCCTGACACGGAAAGAGCTGACTTCATTAG
oIR768	CACATCATAGCGCATGGCGTTTAC
oIR769	CATAAAAGCAGGAAAGCTGAATGTC
oIR770	GGTACTGAGCGAGGGAGCAGAATAAGGCACCTTCTTTTTATTATTCTTTTTAAGTATTGC
oIR783	TAACAATTAAGCTTacataaggaggaactactATGAAACAGTCACAGCCAGTATTAAC
oIR784	CTGCAGttACTAGTTTAACTGTACTTTTTTTCTAACCGCTC
oIR785	TAACAATTAAGCTTacataaggaggaactactATGAGCAGACATATTCAATATTCAATTG
oIR786	CTGCAGttACTAGTTTATGACATATGCTGGTCTCTATAGAG
oIR787	tAATTGTGAGCGGATAACAATTAAGCTTacataaggaggaactactATGTTTTCTATAATTATACCAATATA TAATTCTG
oIR788	CgaGCTAGCatCTGCAGttACTAGTTTACCTTTTTTTAAAAACCCTTGTAAGC
oIR961	CTAGTagcaccggcAGCGGCTCTACTAGTATGACGATCGATGAAATTTACCAAATG
oIR962	tatcacctcaaatggttcgctggGATCCTCATTCATCATTAACACCTCTATTATAAAGTGC
