# Supplementary material for: Bacillus subtilis uses the SigM signaling pathway to prioritize the use of its lipid carrier for cell wall synthesis
Source: PLoS Biol. 2024 Apr 29;22(4):e3002589. doi: 10.1371/journal.pbio.3002589 (PMC11081497; doi:10.1371/journal.pbio.3002589)
Supplement: Attachment — Submitted filename: point-by-point response with reviewer figure (final).pdf [file pbio.3002589.s003.pdf]

From the Editor:
In light of the reviews, which you will find at the end of this email, we would like to invite you to revise the work to thoroughly address the reviewers' reports.

As you will see below, the reviewers agree that this is an interesting work, but further experimentation is needed. In particular, we think it is important to experimentally address the effect of overexpressing UppS, and, although we are aware that the mutant lacking TagO is not viable, the effect of lack of TagO if possible.

Given the extent of revision needed, we cannot make a decision about publication until we have seen the revised manuscript and your response to the reviewers' comments. Your revised manuscript is likely to be sent for further evaluation by all or a subset of the reviewers.

We expect to receive your revised manuscript within 3 months. Please email us (plosbiology@plos.org) if you have any questions or concerns, or would like to request an extension.

At this stage, your manuscript remains formally under active consideration at our journal; please notify us by email if you do not intend to submit a revision so that we may withdraw it.

Reviewer #1: Jörg Stülke. Bacillus subtilis.

Reviewer #1: Roney & Rudner provide a compelling in vivo analysis on the regulation of the YhdLK anti sigma factor via undecaprenylphosphate in B. subtilis. Even though the authors are unable to provide ultimate in vitro data (probably because of the problems intrinsically linked to the work with membrane proteins which preclude such experiments), the data are highly convincing. The paper is well written.

We thank the reviewer for their careful reading of the manuscript for their enthusiasm for our findings.

I have only very few comments:
1) l. 71: "Fosfomycin inhibits MurA": do you mean MurAA and MurAB or one of them?

MurAA is the essential MurA paralog in *B. subtilis* and our depletion strain only depletes MurAA. Throughout the original submission we referred to MurAA as MurA. We thank the reviewer for catching our error. We have modified the text and use MurAA in the revised manuscript.

2) l. 291 ff: The authors mention the broad conservation of sigM-YhdLK in Bacilli. However, there is no COG for YhdL and YhdK. YhdL is present in B. licheniformis, already the orthodox in B. anthracis has only very weak similarity (27.9% conservation), and the protein is absent from other important bacteria of the class Bacilli such as Listeria monocytogenes or Staphylococcus aureus. YhdK is present in B. licheniformis, but not even in B. anthracis not to mention other Bacilli. In conclusion I would use a statement like "SigM-YhdLK is confined to B. subtilis and close relatives of the genus Bacillus"

We have modified this statement as suggested by the reviewer. (Thank you)

3) l. 311 ff: Given the absence of a strong conservation of YhdLK in pathogenic bacteria, I would not see a starting point for antibiotic discovery. Please delete this paragraph.

We agree with the reviewer that SigM or YhdLK would not be a good antibiotic target. However, since the SigM-YhdLK signaling pathway is induced in response to a reduction in the pool of UndP and virtually all antibiotics that target cell envelope biogenesis reduce this pool, a SigM-responsive promoter could be a very effective tool in discovery of new cell envelope targeting drugs. For this reason we request keeping this paragraph in the Discussion. We have clarified this point to address the reviewer's concern It now reads:

SigM signaling as a tool for antibiotic discovery

Although SigM would not be a good antibiotic target, the findings presented here reinforce the idea that small molecule screens based on the activation of a SigM-responsive reporter are a worthwhile strategy to identify compounds that impair virtually all steps in envelope biogenesis. In fact, a SigM activity reporter has been used previously to screen for small molecules that target the cell wall [17, 56]. The hits from this screen were found to specifically target UndP synthesis. Even weak hits from similar screens have the potential to identify novel inhibitors of cell wall synthesis that trap UndP intermediates or impair precursor biogenesis. These could serve as the starting point for medicinal chemistry campaigns to improve potency and selectivity.

Reviewer #2: Bacterial surface.

Reviewer #2: Bacterial surface glycans are assembled on a universal carrier lipid known as undecaprenyl phosphate (Und-P). Und-P is first synthesized as undecaprenyl pyrophosphate (Und-PP) by the UppS synthase. Und-PP is also released from Und-PP-linked intermediates during glycan polymerization. Und-PP is then dephosphorylated to Und-P by integral membrane phosphatases. At this point, the free pool of Und-P is distributed between peptidoglycan (PG) synthesis and multiple non-essential pathways. Thus, Und-P forms a vital regulatory nexus. However, despite its importance, virtually nothing is known about the mechanisms that distribute Und-P. Very little is even known about the events that occur when Und-P becomes limiting. Here, the authors show that in the Gram-positive bacterium Bacillus subtilis, disruptions in Und-P metabolism, either during de novo synthesis or in recycling, trigger signaling through the alternative sigma factor SigM.

Briefly, the authors show that antibiotics that target Und-P, Und-PP, or block release of Und-P from glycans activate the SigM-responsive promoter PamJ. Similar effects were also observed for mutants that trap Und-P in dead-end intermediates. Reducing the rate of Und-P synthesis genetically or chemically also induced SigM signaling. Importantly, antibiotics or mutants not expected to reduce Und-P levels had little effect on SigM signaling. At this point, the authors worked to rule out the possibility that a sugar modification or secondary wall polymer was being sensed by SigM. The authors also showed that overexpressing the uppS synthase (increases Und-P levels) suppresses SigM activation in LTA synthesis mutants. Curiously, LTA synthesis is Und-P independent. Finally, the authors demonstrate that SigM becomes essential when Und-P metabolism is disrupted. This final result suggests that SigM functions to regulate Und-P distribution and has important implications for designing drug combinations.

Overall, the manuscript is well-written, the experiments controlled, and the conclusions mostly appropriate. The idea that SigM prioritizes Und-P distribution in Bacillus is an intriguing finding that should be followed up on. However, since the study does not include any direct Und-P measurements, the authors should include a little more data showing how overexpressing the Und-PP synthase (is known to increase the pool of Und-P) blunts SigM signaling. The authors also need to revisit the

tunicamycin/fosfomycin experiment to rule out indirect effects. That said, the paper is nearly ready.

We thank the reviewer for their careful reading and evaluation of our manuscript. We also thank them for their enthusiasm and indicating that the paper is "nearly ready". We have addressed the concerns raised above with additional experiments. These are described in detail below.

Antibiotics that block Und-P recycling rapidly activate SigM
The authors show that amphomycin, bacitracin, vancomycin, and ramoplanin activate SigM signaling after 30 minutes exposure. Since these antibiotics are expected to disrupt Und-P recycling, the authors "support a model in which SigM is activated by a drop in the pool of UndP and a not a general block to cell wall synthesis." However, the authors do not measure the effect of these antibiotics on Und-P levels and SigM is still triggered (albeit at a lower level) by antibiotics not expected to disrupt Und-P metabolism (Figure S1C). For these reasons, the authors should determine if overexpressing uppS (like in Figure S10) delays/blunts SigM signaling for cells treated with antibiotics that disrupt Und-P recycling.

PART1:
The reviewer indicated that we do not directly measure the pool of UndP. In the original submission, we referenced our previous work (Roney and Rudner, 2022) in which we used fluorescently labeled MX2401 (MX2401-FL) that binds outward-facing UndP to monitor the levels of the carrier lipid in cells treated with vancomycin and in cells overexpressing UppS. In Extended Data Figure 7 in that study, we show that vancomycin reduces the level of outward-facing UndP and that overexpressing UppS increases its level. However, prompted by the reviewers' request, we revisited these experiments with a larger panel of cell wall targeting antibiotics and an analysis of UndP pools on a timescale relevant to the current study. Importantly, we analyzed the entire free pool of UndP by labeling cells with MX2401-FL and duramycin, a cyclic peptide lantibiotic, that permeabilizes the cells enaabling our probe to access the inner leaflet of the membrane. This method is documented in our original paper (Roney and Rudner, 2022).

The new results nicely mirror our SigM reporter activity data and the release of GFP-SigM from the membrane and its association with the nucleoid. Specifically, we observed a rapid (<2 min) drop in the free pool of UndP after exposing cells to drugs (vancomycin, bacitracin, ramoplanin) that trap UndP intermediates. These same drugs rapidly induce SigM activity and rapidly (<2 min) release GFP-SigM from the membrane. By contrast, fosfomycin that only indirectly traps UndP through the accumulation of UndP-WTA had no impact on the UndP pool after 2 minutes. However, after exposure to fosfomycin for 20 minutes, the UndP pools were markedly decreased. These results are fully consistent with our experiments using the SigM activity reporter and GFP-SigM localization. We have included these new results in **Figures 1F and S3**. We think these additional experiments significantly strengthen our conclusions by directly linking UndP levels to SigM activiation. We thank the reviewer for requesting them.

In addition, we also used fluorescently labeled MX2401 (MX-FL) to compare the free pool of UndP in cells treated with fosfomycin, tunicamycin, and both antibiotics. The data in the original submission showed that SigM is activated albeit slowly by fosfomycin but this activation is largely suppressed by tunicamycin. Using MX-FL, we find the UndP levels drop after ~20min in the presence of fosfomycin and this can be largely suppressed by tunicamycin. Again, there is a nice correlation between UndP levels and SigM activtation. We have included these new data in **Figures 2D and S5**. We think these results significantly strengthen our conclusions and thank the reviewer for prompting us to measure UndP levels.

From comments above: For these reasons, the authors should determine if overexpressing uppS (like in Figure S10) delays/blunts SigM signaling for cells treated with antibiotics that disrupt Und-P recycling.

PART2:
This reviewer also requested we determine whether overexpressing UppS (thereby increasing the pool of UndP) can delay or blunt SigM signaling of cells treated with antibiotics that disrupt UndP recycling.

We had previously performed this experiments with results that were consistent with our model. However, we did not include the data in our original submission because of the challenges in interpreting these experiments.

Specifically, we analyzed SigM activity after exposure to vancomycin in wild-type cells and separately in cells overexpressing UppS. As anticipated, SigM activity was lower in cells overexpressing UppS. This is consistent with a slower drop in UndP levels causing a slower activation of SigM. However, an alternative interpretation of these data that we could not exclude is that increasing the pool of UndP (by overexpressing UppS) increases the pool of lipid II (the UndP-linked PG precursor). Since vancomycin binds lipid II, higher levels of lipid II would also be predicted to slow SigM activation. This difficulty in interpretation exists for all antibiotics that trap UndP-linked products or prevent its recycling. We have included a reviewer figure in the resubmission that reports our findings with vancomycin. However, because of the ambiguity in the interpretation, we have not included the data in the final version of the paper. Instead, as indicated above, we now include data on the impact of these drugs on UndP levels using fluorescently labeled MX2401 (MX-FL). We think these experiments more convincingly address the concerns raised by the reviewer. We hope you agree.

Inhibition of WTA synthesis suppresses fosfomycin activation of SigM.
The authors argue that fosfomycin, which does not directly induce Und-P sequestration, triggers SigM signaling due to the accumulation of Und-PP-linked WTA precursors (are attached to PG). To test this hypothesis, the authors simultaneously treated their cells with fosfomycin and tunicamycin, which inhibits the committing step in WTA synthesis. As expected, SigM activation was reduced. However, tunicamycin also inhibits MraY (PG synthesis). Thus, the fosfomycin/tunicamycin results likely represent a mixture of causes. Therefore, recommend testing the effect of fosfomycin on SigM activation in a mutant lacking TagO, which initiates WTA synthesis.

Tunicamycin inhibits the committing enzyme (TagO) in the WTA precursor synthesis pathway but also the enzyme (MraY) that attaches MurNAc-pentapeptide to UndP. Fosfomycin inhibits the committing enzyme (MurAA) in PG precursor synthesis, upstream of MraY in the lipid II synthesis pathway. As such, fosfomcyin blocks the production of MraY's substrate (UDP-MurNAc-pentapeptide). Accordingly, tunicamycin-mediated inhibition of MraY in the presence of fosfomycin should have minimal impact on SigM activation.

However, to avoid any possible off-target affects, we repeated these experiments with a concentration of tunicamycin (2 µg/mL) that inhibits TagO but not the essential enzyme MraY. We compared SigM activity using our P(amj)-yfp reporter in the presence of fosfomycin and separately in the presence of fosformcyin and 2 µg/mL tunicamycin. SigM activity was suppressed in the presence of these lower

levels of tunicamycin and looked indistinguishable from the data in the original submission using 34 µg/mL tunicamycin. We have included this control as a supplemental figure (**Fig. S4**) in the resubmission.

Minor

Lines 14-15: "UndP is maintained at low levels in the cytoplasmic membrane (~10^5 UndP/molecules/cell)." Could the authors explain why they think 100,000 molecules is low? What level would be considered high?

~10^5 UndP/molecules/cell represents approximately 0.1% of all membrane lipids (ref 8). This level is generally considered low in comparison to how much it is utilized during growth. For example, just for peptidoglycan synthesis, >10^6 molecules of lipid II are used every cell cycle. UndP is also used for WTA synthesis and surface modification. To address this concern, we have modified the text to indicate that UndP is present at ~0.1% of all membrane lipids and included the reference above.

Line 97: "tunicamycin reduced fosfomycin-mediated SigM activation at both 30 and 60 minutes (Fig. 2AB)." The authors should incorporate a concluding statement after this statement.

Thank you for requesting this. We now include the data described above where we assess the free pool of UndP after exposure to fosfomycin, tunicamycin, and both. The end of this paragraph now reads:

Strikingly, tunicamycin reduced fosfomycin-mediated SigM activation at both 30 and 60 minutes (**Fig. 2AB and S4**). Furthermore, UndP levels, as assayed by MX2401-FL, were reduced after exposure to fosfomycin for 20 min, consistent with the observed increase in SigM activity at 30 and 60 min. Importantly, tunicamycin largely suppressed the drop in UndP levels caused by fosfomycin (**Fig. 2D and S5**). Thus, all drugs that target cell wall synthesis directly or indirectly reduce the free pool of the carrier lipid, consistent with the model that the YhdLK-SigM complex is responsive to UndP levels.

Lines 154-155: "the primary source of UndP for PG synthesis is the recycled lipid carrier and new UndP synthesis principally maintains the pool size during growth." Really? How would the cell distinguish between these two pools?

We thank the reviewer for highlighting this confusing sentence.

During the cell cycle, the cell must increase the number of UndP molecules 2-fold such that upon cytokinesis each daughter cell receives ~10^5 molecules. Thus, during the cell cycle the larger share of precursors are built on the recycled material. In responding to the reviewer's query, we realized this is not the point we intended to make. We have modified this sentence as follows:

We note that fosmidomycin took longer to impair growth compared to the antibiotics that target cell wall synthesis (**Fig. S9C**). This finding is consistent with the idea that the recycled lipid carrier can sustain growth for a period without the production of new UndP molecules to maintain the pool size as the cell elongates.

Lines 206-208: "Our model predicts that the overexpression of CsbB in the absence of YfhO activates SigM due to sequestration of UndP in UndP-GlcNac. If correct, the addition of tunicamycin and fosfomycin during overexpression of CsbB should have no impact on the activation of SigM." However, the authors contend that tunicamycin inhibits WTA synthesis (line 96). Since WTAs synthesis "requires a significant share the carrier lipid pool," inhibiting WTA synthesis would be expected to increase Und-P levels and potentially overcome the effects of Und-P sequestration.

The strong CsbB overexpression used in this experiment likely depletes UndP so rapidly and efficiently that it would be difficult for tunicamycin to suppress the SigM activity.

However, prompted by the reviewer's query, we repeated this experiment overexpressing CsbB to a lower level leading to a lower level of SigM activation. Even under these conditions tunicamycin did not reduce SigM activity. This suggests that WTA synthesis uses a relatively small portion of the UndP pool. Even though WTA can make up 50% of the dry weight of the envelope, each WTA polymer contains multiple glycerol-phosphate repeats that are all built on a single carrier lipid. So, a small number of UndP molecules could be engaged in WTA synthesis at any time. Alternatively, the rate of WTA synthesis might be very high such that a small number UndP are actively engaged in WTA synthesis at any given time. In either case, addition of tunicamycin would liberate a relatively small amount of UndP.

We contrast this to tunicamycin's ability to suppress SigM activation caused by fosfomycin treatment. Since there is no nascent PG to transfer the WTA, UndP-linked WTA precursors accumulate (and trap UndP). Under these conditions many UndP molecules would be sequestered. In other words, tunicamycin isn't liberating UndP, in this experiment instead it is preventing UndP-WTA accumulation.

Based on these follow up experiments, we have deleted a sentence from the original submission that suggested WTA synthesis requires a significant share of the carrier lipid pool. This was not critical to our argument and we are no longer certain this is the case.

In the original submission we wrote:
"WTAs make up ~50% of the dry weight of the *B. subtilis* cell wall and therefore its synthesis requires a significant share the carrier lipid pool [25]."

In the revision, we removed this sentence. It does not change any of our conclusions. As indicated above, our findings do not require that a significant share of the carrier lipid pool is used to produce WTAs under unperturbed conditions - just that UndP-WTAs accumulate (and trap UndP) when they can't be transferred to the nascent PG.

Lines 237-241: This information should be included in the introduction, somewhere around Line 25, to reinforce the argument that SigM prioritizes Und-P for PG synthesis.

We now include this information. The sentence now reads:
Transcriptional profiling experiments revealed that many of the genes in the SigM regulon are involved in cell wall biogenesis [12, 13]. Specifically, SigM controls several genes involved in the synthesis and transport of PG precursors as well as PG polymerization and crosslinking. SigM also regulates genes involved in recycling UndP, its de novo synthesis, and liberation of the carrier lipid from UndPP-WTA precursors.

Figure 7B and 7C: The dilution factor should be noted on the figure or in the legend.
Is there a reason the authors did not include scale bars on their micrographs?

We now included dilution factors and scale bars.

Other
Figure 1A: "Antibiotics used in this study are shown in red." Should the authors include tunicamycin?
Figure S3: "Stains depleted" to "Strains depleted"
Line 91: "requires a significant share the carrier lipid" to "requires a significant share of the carrier lipid"

Figure 1A: We do not include tunicamycin in this figure because the schematic is meant to focus the reader on the synthesis of peptidoglycan. We now mention tunicamycin in the figure legend.

Figure S3: corrected (thank you)

Line 91: we have modified this sentence (see above).

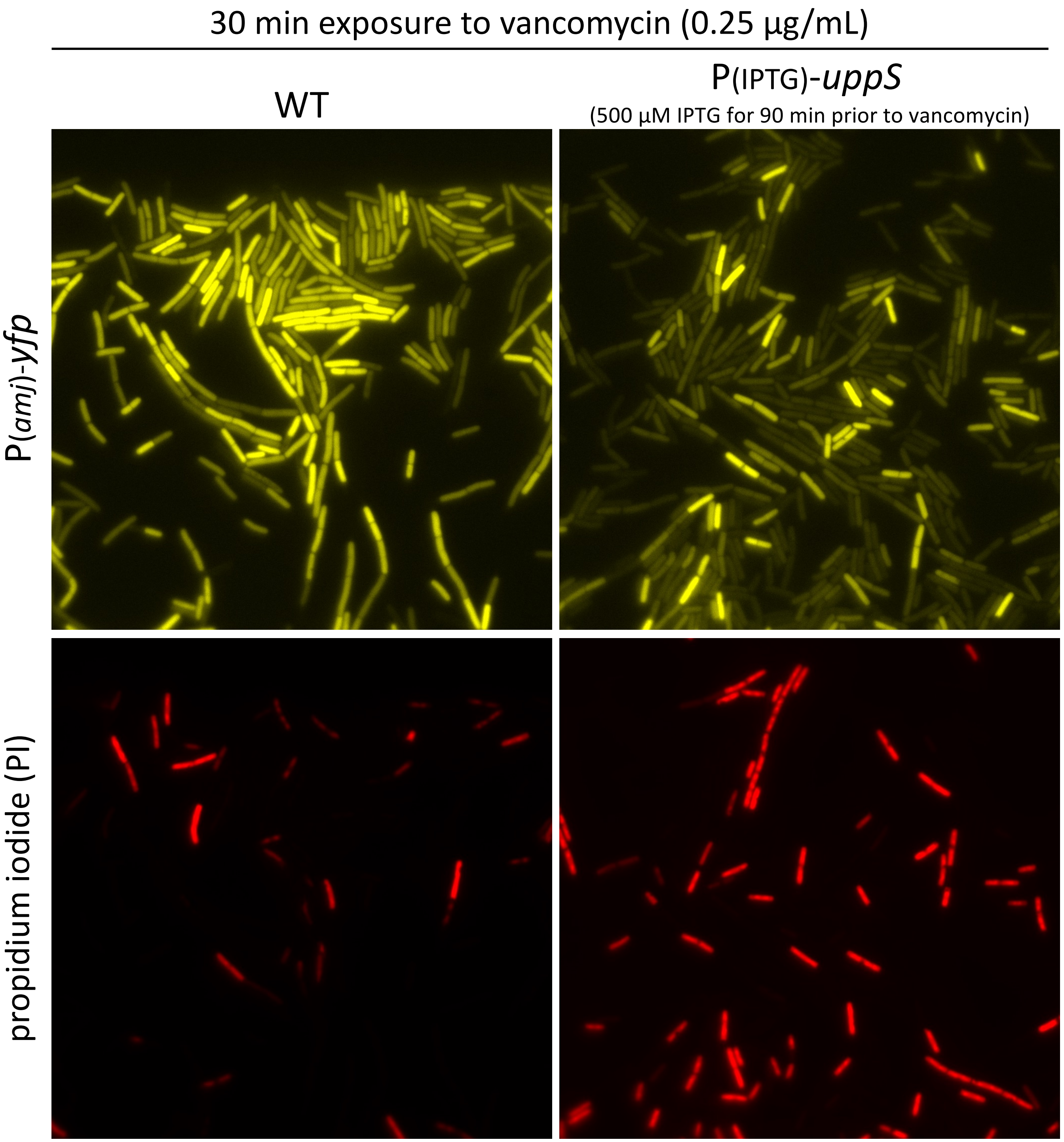

Reviewer Figure: Overexpression of UppS partially suppresses SigM activation in response to vancomycin. Representative fluorescence images of the indicated strains after exposure to subinhibitory concentrations of vancomycin (2.5 µg/mL) for 30 min. Both strains harbor the SigM-responsive reporter P(*amj*)-*yfp*. The strain with the IPTG-regulated allele of *uppS* was grown for 90 min in the presence of saturating concentrations of IPTG to increase UndP levels prior to vancomycin addition.
